# *Leishmania infantum* Induces the Release of sTREM-1 in Visceral Leishmaniasis

**DOI:** 10.3389/fmicb.2017.02265

**Published:** 2017-11-16

**Authors:** Lays G. S. Bomfim, Lucas S. Magalhães, Marcello A. A. Santos-Filho, Nalu T. A. Peres, Cristiane B. Corrêa, Diego M. Tanajura, Angela M. Silva, Michael W. Lipscomb, Valéria M. Borges, Amélia R. Jesus, Roque P. Almeida, Tatiana R. de Moura

**Affiliations:** ^1^Laboratório de Biologia Molecular-Hospital Universitário, Universidade Federal de Sergipe-Aracaju, Sergipe, Brazil; ^2^Department of Biology, Howard University, Washington, DC, United States; ^3^Instituto Gonçalo Moniz, Fundação Oswaldo Cruz, Salvador, Brazil

**Keywords:** visceral leishmaniasis, *Leishmania infantum*, neutrophils, sTREM-1, TREM-1

## Abstract

Visceral leishmaniasis (VL) is a systemic transmissible disease that remains to be a major global health problem. The inflammatory response during VL is characterized by the release of several cytokines and other pro-inflammatory mediators. Triggering Receptor Expressed on Myeloid Cells (TREM) are a group of evolutionarily conserved membrane-bound surface receptors expressed on neutrophils and monocytes. Engagement of TREM-1 directs intracellular signaling events that drive cytokine production, degranulation, and phagocytosis. In certain inflammatory-associated diseases, TREM-1 can also be found as a soluble form (sTREM-1), which can negatively regulate TREM-1 receptor signaling. In these studies, we now find that high levels of circulating sTREM-1 correlate directly with VL disease severity. In particular, high levels of sTREM-1 were observed in non-survivor VL patients. Furthermore, these levels of sTREM-1 positively correlated with liver size and negatively correlated with leukocyte counts and hemoglobin concentration. Moreover, we found that neutrophils exposure *in vitro* to *Leishmania infantum* modulates TREM-1, DAP12, and IL-8 gene expression, while also increasing release of sTREM-1. Finally, results revealed that higher sTREM-1 levels are associated with increasing parasite ratio. Taken together, these studies suggest that *L. infantum* may modulate TREM-1 in neutrophils and high levels of this molecule is associated with severe VL.

## Introduction

Visceral Leishmaniasis (VL), a neglected tropical disease in the Americas, is caused by the *Leishmania infantum* parasite. VL exhibits a broad spectrum of clinical manifestations that vary from asymptomatic to the classic pathological disease state. The latter can be characterized by long-term fever, hepatosplenomegaly, anemia, leucopenia, thrombocytopenia, and weight loss. If not treated in a timely manner, this classic pathological disease can result in severe morbidity and/or cause mortality (Sampaio et al., 2010).

Neutrophils, as the most abundant leukocytes in humans, serve a pivotal role in combating and defending against invading pathogens. However, their role in VL is poorly understood. Recently, it was shown that circulating neutrophils were highly activated in patients with VL in Ethiopia, but that their pro-inflammatory effector functions were impaired (Yizengaw et al., 2016). Several receptors displayed on the surface of neutrophils can recognize foreign molecules and serve to initiate pro-inflammatory response against microorganisms. One particularly novel family of evolutionary conserved receptors are the Triggering Receptor Expressed on Myeloid Cells (TREM) molecules, which are expressed in neutrophils, macrophages, and in subsets of monocytes (Bouchon et al., 2000).

In particular, TREM-1 expression can be directly induced by engagement with microbial products. Interaction with cognate ligands has shown to trigger release of IL-8, TNF-α, IL-1β, MIP-1α, and MCP-1 pro-inflammatory cytokines (Bouchon et al., 2000; Arts et al., 2013). Interestingly, TREM-1 can also be found in its soluble form (sTREM-1), which seems to negatively regulate TREM-1 receptor signaling (Gibot et al., 2004b; Klesney-Tait et al., 2006). Although the origin of native soluble TREM-1 remains controversial, the potential role of sTREM-1 in regulating infectious diseases has been extensively investigated (Gibot et al., 2004a; Cao et al., 2017). Many studies point to the potential use of sTREM-1 as a biomarker for infectious and non-infectious inflammatory diseases, such as sepsis (Gibot and Cravoisy, 2004) and rheumatoid arthritis (Molad et al., 2015). In cases of VL, biomarkers that can adequately assess disease severity would improve the clinical monitoring of patients.

In the present study, we investigated sTREM-1 serum levels in VL patients and membrane-bound TREM-1 receptor expression on neutrophils exposed to *L. infantum*. Our results show that high levels of sTREM-1 are present in VL patients, with the highest levels in non-survivors of the disease. We found that sTREM-1 positively correlated with increased liver size and negatively correlated with leukocyte counts and hemoglobin concentration. Corroborative *in vitro* studies showed that *L. infantum* infections into neutrophils directly induced TREM-1, DAP12, and IL-8 gene expression. Additionally, there was a significant increase in sTREM-1 release and concomitant decrease of membrane-bound TREM-1 from these infected neutrophils. Taken together, the results suggest an important role of sTREM-1 and presents a case for use as a biomarker to evaluate disease severity.

## Materials and methods

### Ethics statement

This study was approved by the Ethics Committee of the University Hospital of the Federal University of Sergipe (CAAE-53366916.8.0000.5546). All clinical investigations were conducted with informed consent obtained from all participants or legal guardians, in accordance with the Declaration of Helsinki.

### Subjects and sample collection

The study assessed individuals in five groups: (1) patients with classical VL manifestation before leishmaniasis chemotherapy, day 0 (D0) (*n* = 21); (2) patients with classical VL manifestation 30 days after treatment (D30) (*n* = 16); (3) patients with severe VL (SVL) before leishmaniasis chemotherapy (*n* = 10); (4) DTH+ are subjects with positive delayed type hypersensitivity (DTH) skin test, who did not develop VL (*n* = 10); (5) healthy controls (HC) are subjects without infectious or other inflammatory diseases (*n* = 17). The patients were recruited at the Reference Center at the University Hospital in Sergipe, Brazil, diagnosed with VL, and the patients with SVL were classified based on clinical score described by Sampaio et al. (2010). Furthermore, these individuals were validated by a new more complete criteria that has both higher sensitivity and specificity (Costa et al., 2016). Blood was collected from all subjects. Sera was processed and stored at −80°C until use. For the *in vitro* experiments, peripheral blood from healthy human donors was used.

### *Leishmania infantum* parasites

The parasite *L. infantum* (MHOM/BR/2010/LVHSE49) used in this study was obtained from a patient with VL in 2010 in Aracaju, Brazil. The parasite was isolated by bone marrow puncture (before the start of the therapeutic regimen) and inoculated into Novy, Mac Neal and Nicole (NNN) and Schneider's Insect medium (Gibco, NY, USA) supplemented with 10% fetal bovine serum (Sigma-Aldrich Co., MO, USA) and 1% penicillin/streptomycin. Parasites were kept in frozen stocks after only one passage in culture to limit expansion of mutant strains. *Leishmania* isolates were expanded in supplemented Schneider's medium at 24°C. Promastigotes were examined daily using light microscopy to determine growth curves.

### Neutrophils culture and exposure to *L. infantum*

Neutrophils were isolated from the peripheral blood of healthy human donors using Polymorphprep™ (Axis-Shield). After isolation, neutrophils were re-suspended in RPMI 1640 culture medium (Gibco, Carsbad, CA, USA) supplemented with 1% nutridoma and 1% penicillin/streptomycin. The cells were diluted to a concentration of 2.5 × 10^6^ cells/ml and plated into 96-well plates in a total volume of 200 μl of complete RPMI-1640 medium (5 × 10^5^ cells/well). For infection, neutrophils were exposed to *L. infantum* promastigotes in the stationary phase (MHOM/BR/2010/LVHSE49) at ratios of 1:1, 5:1, or 10:1 (parasites:neutrophil). Cells and parasites were then cultured for 30 min or for 3, 6, or 9 h at 37°C in 5% CO_2_. After the exposure period, supernatants were collected and stored at −80°C until use.

### Quantitative real-time polymerase chain reaction (qPCR)

To evaluate gene expression after neutrophil exposure to *Leishmnia*, cells were harvested and resuspended in Trizol reagent (Life Technologies) prior to total RNA extraction. Total RNA was reverse transcribed into single-stranded cDNA using the High Capacity cDNA Reverse Transcription Kit (Applied Biosystems). For real time PCR reactions, the Applied Biosystems TaqMan Gene Expression Master Mix and Gene Expression Assay kits were used (assay IDs TREM-1: Hs00218624-m1, DAP12: Hs00182426_m1, IL-8: Hs00174103_m1). Expression levels of the target transcripts were calculated by the comparative Ct method (2^−ΔΔCt^ formula) after normalization with the house-keeping gene GAPDH (Hs99999905_m1).

### Flow cytometric analysis for TREM-1 detection on the neutrophils surface

To evaluate the surface expression of TREM-1 on neutrophils, *L. infantum* parasites were pre-labeled with fluorescent probe CellTracker™ Violet BMQC (Life Technologies) before adding to neutrophils, following the protocol previously described (Dagley et al., 2015). After 30 min or 3 h, exposed cells were washed and incubated with anti-CD354/TREM-1 (Biolegend). Data was acquired using the FACS CANTO II (BD Biosciences) and analyzed using FlowJo v10.0 software (Tree Star).

### Measurement of sTREM-1 by ELISA

The levels of sTREM-1 in the serum and cell-free culture supernatants were measured by specific enzyme-linked immunosorbent assay (ELISA) kits (DuoSet, R&D Systems, Abingdon, UK). The absorbance at 450 nm was measured using a microplate reader (Epoch, BioTek, Luzern, Switzerland), with a wavelength correction set at 570 nm to subtract background. A standard curve was generated for each set of samples assayed using manufacturer's recommended protocol.

### Statistical analysis

Median and interquartile ranges were used as measures of central tendency for the *ex vivo* analyses. Mean and standard errors were used to display data from the *in vitro* experiments. Differences between two groups were calculated using the Student *t*-Test, Mann–Whitney *U-*test, or the Kruskal-Wallis test with the Dunn. Multiple comparisons or linear trend analysis post-tests (more than 2 groups) used Friedman with Bonferroni post-test and One-Way ANOVA. Correlations were tested using Spearman and Pearson ranks. Differences with *p* < 0.05 were considered statistically significant. Analyses were performed using Prism 6.0 software (GraphPad).

## Results

### High serum levels of sTREM-1 was associated with VL severity

**Figure 1:**
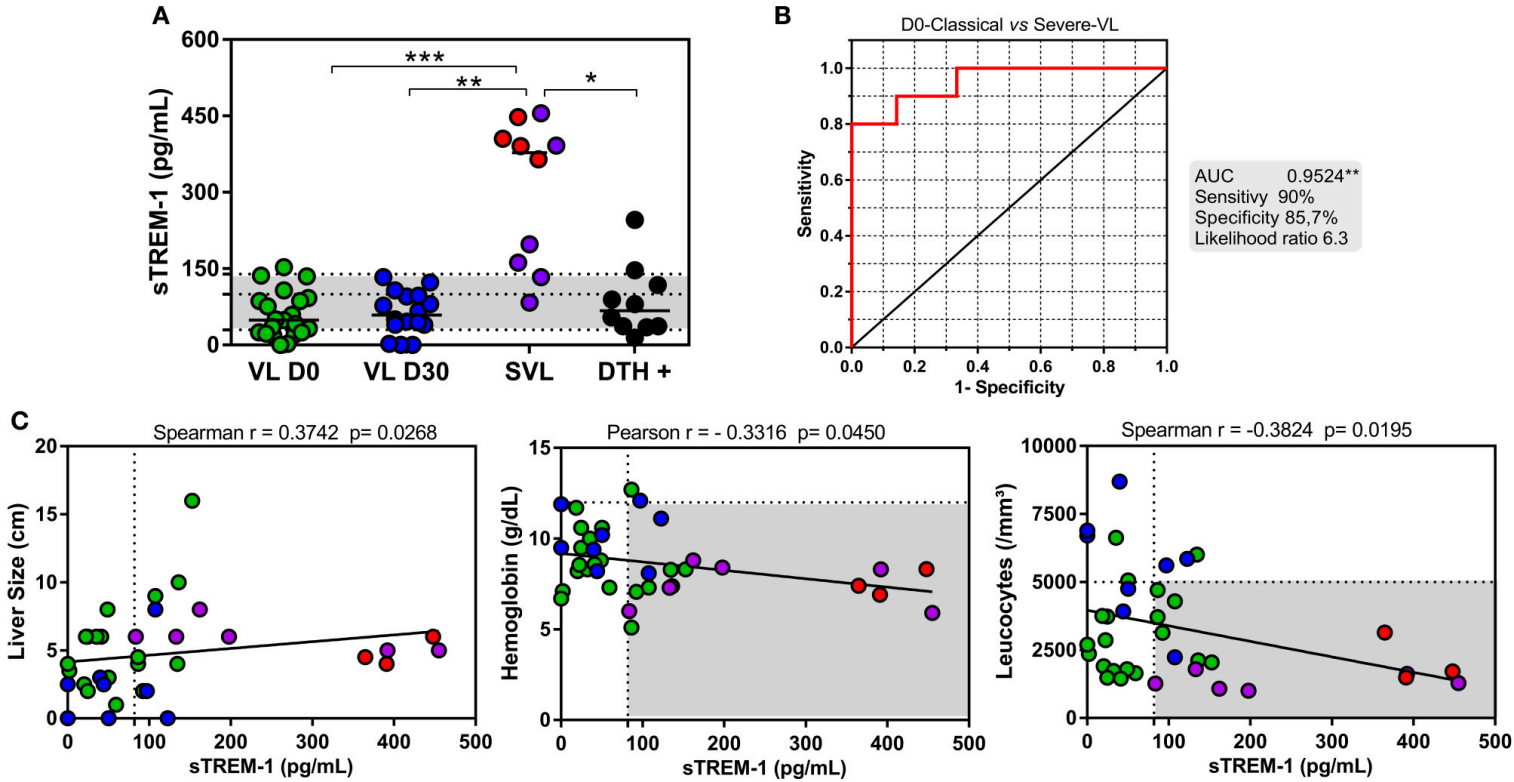
sTREM-1 serum levels were associated with severe visceral leishmaniasis (SVL). **(A)** sTREM-1 measured in serum samples from classical VL patients before (VL D0; green; *n* = 21) and after treatment (VL D30; blue; *n* = 16); patients with severe VL (SLV) that survived (purple; *n* = 6) and those that died (red; *n* = 4); DTH+ subjects (black; *n* = 10). Shaded region represents median and interquartile range values of sTREM-1 from 17 healthy endemic controls to serve as reference (^*^P < 0.05, ^**^P < 0.01, ^***^P < 0.001, by ANOVA and Bonferroni post-test). **(B)** A receiver operating characteristic (ROC) curve was generated for discriminates the levels of sTREM-1 between patients with classical VL from patients with severe VL (area under the ROC curve [AUC], 0.9524; *P* = 0.0001). **(C)** Correlations between serum sTREM-1 levels with liver size, leukocyte counts and hemoglobin levels in patients with VL and SVL. Dotted lines on the X-axis represent the median value of sTREM-1 of the healthy endemic controls. Dotted lines on Y-axis indicate cell count of respective clinical parameter. Shaded region designate individuals simultaneously displaying sTREM-1 levels below the medians and clinical parameters. Correlations were analyzed using Spearman and Pearson ranks.

Serum levels of sTREM-1 were measured in patients with distinct VL clinical forms. Results revealed higher levels of sTREM-1 in severe VL (SVL) patients compared to all groups (*P* < 0.05) (Figure 1A). The median level of sTREM-1 among SVL patients (377.9 pg/ml) was about 7-fold higher than that among patients with classic VL D0 (48.77 pg/ml). This was 6.45-fold higher than classical VL D30 and 5.61-fold higher than DTH+. Interestingly, all non-survivor SVL patients showed sTREM-1 levels above 300 pg/ml. Furthermore, 90% of the SVL group showed levels above the 99.4 pg/ml found within the control group. In fact, by use the receiver-operating characteristic (ROC) curve, serum-sTREM-1 levels had 90% sensitivity and 85.7% specificity for differentiating patients with classical VL D0 from those SVL, with the cut-off value of 120.3 pg/ml and likelihood ratio of 6.3 (area under the ROC curve, 0.9524; *P* = 0.0001), authenticating its clinical utility as a biomarker to discriminate the severity form (Figure 1B). To investigate whether increased sTREM-1 levels are associated with disease, we correlated sTREM-1 levels with liver enlargement, leukocyte counts and hemoglobin concentration (Figure 1C). Liver enlargement, which is the classical manifestation of VL, positively correlated with circulating sTREM-1. Leukocyte counts and hemoglobin concentration negatively correlated with sTREM-1. Interestingly, SVL patients showed high sTREM-1 levels and lower counts of lymphocytes and neutrophils (Table 1).

**Table 1:** Clinical data of patients with classical visceral leishmaniasis (VL) and severe (SVL) in the pre-treatment phase.

**Parameters**	**Patients with VL (D0) (*n* = 21)**	**Patients with SVL (*n* = 10)**	**Value de *p***
Gender (M/F)	11/10	9/1	–
Age	17.43 ± 16.77	26.80 ± 21.97	0.1986
Spleen (cm)	8.094 ± 3.908	8.167 ± 3.162	0.9448
Liver (cm)	5.417 ± 3.611	5.611 ± 1.167	0.3487
Leucocytes/mm^3^	3,155 ± 1,542	1,599 ± 640.1	0.0074^**^
Neutrophils/mm^3^	1,059 ± 614.0	522.3 ± 277.1	0.0191^*^
Eosinophils/mm^3^	19.49 ± 39.04	3.44 ± 6.579	0.1213
Lymphocytes/mm^3^	1,763 ± 1,362	808.0 ± 634.0	0.0425^*^
Monocytes/mm^3^	288.0 ± 233.4	203.9 ± 115.7	0.3433
Hemoglobin (g/dL)	8.603 ± 1.821	7.479 ± 1.063	0.0979
Hematocrit (%)	26.83 ± 5.398	23.20 ± 3.496	0.0770
Platelets/mm^3^	123,995 ± 83,215	85,566 ± 62,118	0.2294
AST (U/L)	142.4 ± 123.2	143.4 ± 133.6	0.8262
ALT (U/L)	85.5 ± 79.90	1,082 ± 3,302	0.2051

*Values express the mean ± standard deviation. The p-value expresses the statistical difference for clinical outcomes between the two groups of VL patients, analyzed using Student's T-test for parametric data and Mann-Whitney test for non-parametric data*.

* P < 0.05;

** *P < 0.01*.

### Gene expression of TREM-1, DAP12, and IL-8 is increased in neutrophils exposed to *L. infantum*

**Figure 2:**
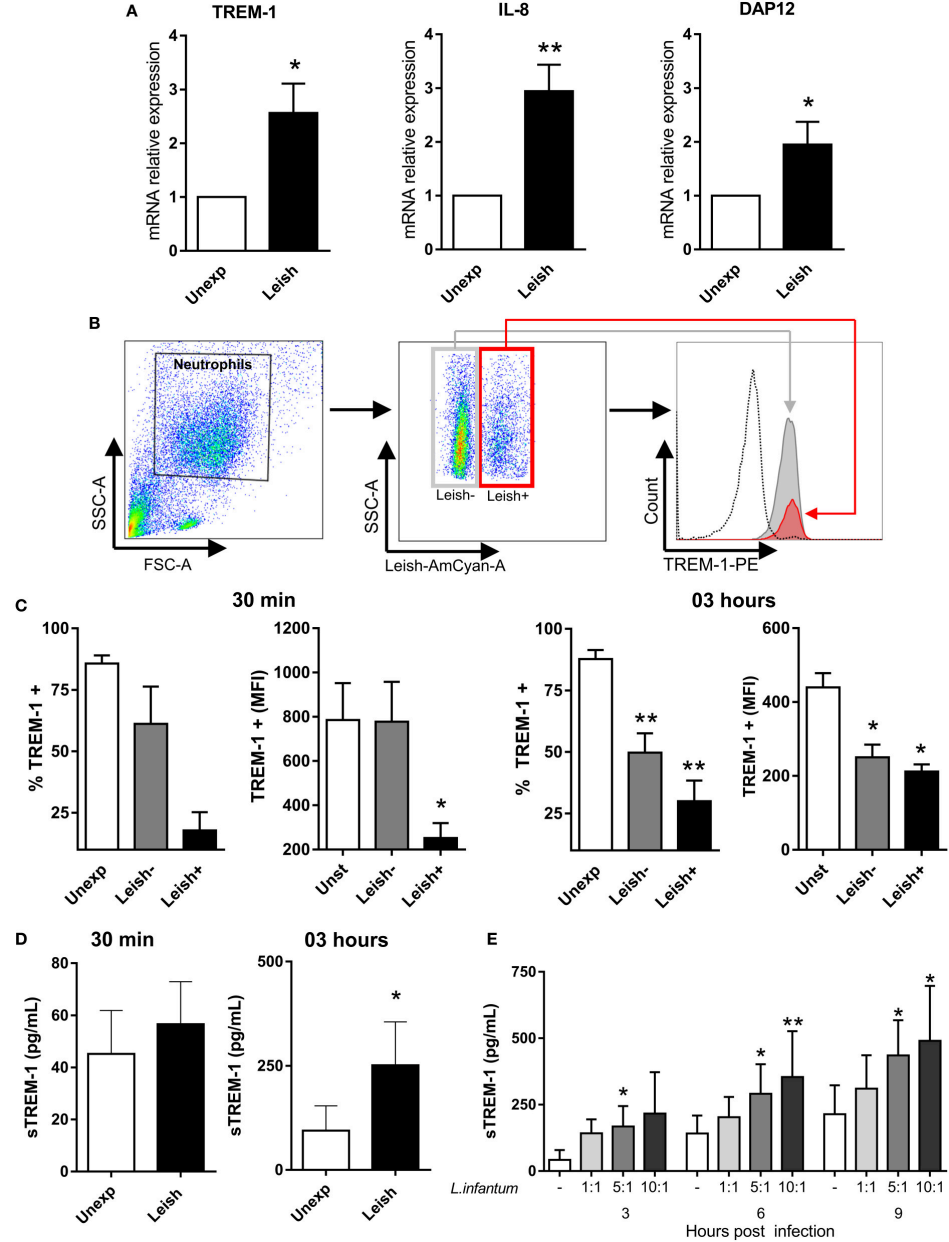
*Leishmania infantum* modulated TREM-1 in human neutrophils. **(A)** Gene expression of TREM-1, DAP12, and IL-8 in neutrophils upon exposure to *L. infantum*. Neutrophils from healthy donors were exposed to *L. infantum* at a 5:1 ratio (parasites:cell). Control group was unexposed neutrophils. Relative gene expression was determined after 30 min of exposure. Bars represent the mean ± standard error of 3 independent experiments with 7 donors (^*^P < 0.05; ^**^P < 0.01, by Student's- *t*-test). The asterisk represents statistically significant difference between the unexposed control and the *L. infantum*-challenged group. **(B)** Gating strategy showing neutrophil region (FSC vs. SSC) with or without *L. infantum* infection (labeled with CellTracker) expressing TREM-1. The dotted line represents the negative cells for TREM-1. **(C)** Neutrophils from healthy donors were exposed to *L. infantum* labeled with CellTracker at a 5:1 ratio (parasites:cell) for 30 min and 3 h. Frequency of neutrophils expressing TREM-1 and MFI was assessed. Bars represent the mean ± standard error of 2 independent experiments with 5 donors (30 min) and 6 donors (3 h) (^*^P < 0.05, ^**^P < 0.01, by Student's- *t*-test). **(D)** Neutrophils from healthy donors were exposed to *L. infantum* at a 5:1 ratio (parasites:cell). After 30 min and 3 h, release of sTREM-1 by neutrophils into the culture supernatants were measured by ELISA. Bars represent the mean ± standard error of 2 independent experiments with 5 donors performed in triplicate (^*^P < 0.05, by Student's- *t*-test). **(E)** Neutrophils from healthy donors were exposed to *L. infantum* at a 1:1, 5:1, and 10:1 ratio (parasites:cell). After for 3, 6, and 9 h, release of sTREM-1 by neutrophils into the culture supernatants were measured by ELISA. Bars represent the mean ± standard error of 2 independent experiments with 5 donors performed in triplicate (^*^P < 0.05; ^**^P < 0.01, by Friedman test with Bonferroni post-test). The asterisk represents statistically significant difference between the unexposed control and the *L. infantum*-challenged groups.

Since VL is largely caused by the *L. infantum* strain, we next investigated whether exposure can modulate TREM-1 expression in neutrophils. Upon infection of peripheral blood-isolated neutrophils with the parasites for 30 min *in vitro*, gene expression of TREM-1, DAP12, and IL-8 was found to be higher compared to unexposed neutrophils (Figure 2A). TREM-1 expression among exposed neutrophils was 2.5-fold higher than the unexposed neutrophils. Similarly, DAP12 and IL-8 expression among exposed neutrophil were higher, with 2.0- and 3.0-fold, respectively, than the unexposed group.

### *Leishmania infantum* induces release of TREM-1 on neutrophils

Next, we evaluated whether neutrophil surface expression of TREM-1 could be a source of sTREM-1 after *L. infantum* exposure (Figures 2B,C). The unexposed group served as internal controls and showed highest frequency and MFI of membrane-bound TREM-1 expression compared to neutrophils infected by *L. infantum* (Leish^+^ population) at both 30 min and 3 h post-exposure. Leish^+^ population had lower frequency and MFI of TREM-1 expression compared to the exposed neutrophils, but not infected (Leish^−^ population) at 30 min post-exposure. In addition, the Leish^−^ population also showed lower frequency and MFI of membrane-bound TREM-1 expression on neutrophils after 3 h, compared to unexposed neutrophils.

To corroborate our findings that high levels of serum sTREM-1 are associated with VL severity, we evaluated the presence of sTREM-1 released in the culture supernatant of peripheral-blood neutrophils infection with leishmania (Figure 2D). Although there was no statistical difference at 30 min of exposure, we observed a slightly increased release of sTREM-1 in the neutrophil exposed to *L. infantum* at a rate of 5:1 (parasites: neutrophils), when compared to the unexposed group. However, at 3 h of exposure, the levels of sTREM-1 in exposed neutrophils were 2.67-fold higher than in the unexposed neutrophils group (*p* = 0.0313). In addition, we assessed whether release of sTREM-1 by neutrophils was associated with increased number and time of *L. infantum* challenge (Figure 2E). For this, neutrophils were exposed to *L. infantum* at a rate of 1:1, 5:1, and 10:1 parasites to neutrophils, respectively, for 3, 6, and 9 h. Results revealed a direct correlation between increased parasite load and exposure time with sTREM-1 release. Taken together, this study shows that *L. infantum* exposure enhances the transcription of TREM-1 and molecules associated to its activation, as well as release of sTREM-1 from neutrophils.

## Discussion

In VL, high serum levels of cytokines play an important role in pathogenesis and can be associated with clinical disease manifestations that may lead to death (Santos et al., 2016; Silva et al., 2017). This is the first report to show that high serum levels of sTREM-1 in patients correlate with severe disease outcomes. We also demonstrate that *in vitro L. infantum* exposure upregulates TREM-1, DAP12, and IL-8 gene expression in neutrophils, while reducing expression of membrane-bound TREM-1 and inducing release of sTREM-1 in the supernatant.

We observed that all SVL patients who died presented high serum level of sTREM-1. Furthermore, there was a correlation between sTREM-1 and clinical markers of disease, such as positive correlation with liver size and negative correlations with both leukocyte count and hemoglobin concentration. Hematological changes are constant in VL, and in untreated cases, patients die due to excessive bleeding, severe anemia, and bacterial infections (Sampaio et al., 2010). In hemodialysis patients, sTREM-1 levels were significantly increased, whereas a negative correlation between hemoglobin concentration and sTREM-1 level was observed (Essa and Elzorkany, 2015). This suggests sTREM-1, together with other clinical and laboratorial parameters can, serve as a novel and valuable biomarker for severity in VL.

sTREM-1 has been also described as an important predictor and diagnostic marker for other infections (Gibot and Cravoisy, 2004). For instance, it was reported that sTREM-1 is valuable for differentiating sepsis from SIRS (Gibot et al., 2004c; Richeldi et al., 2004; Siranović et al., 2011). Moreover, sTREM-1 is described to be valuable for early diagnosis and severity of neonatal sepsis (Adly et al., 2014; Arízaga-Ballesteros et al., 2015; Saldir et al., 2015). However, a systematic review and meta-analysis shows that sTREM-1 had a moderate diagnostic performance in differentiating sepsis from SIRS in adult patients (Wu et al., 2012). Additionally, sTREM-1 alone is insufficient as a biomarker to predict mortality (Su et al., 2016).

Interestingly, we demonstrated that the exposure to *L. infantum* increased TREM-1, DAP12, and IL-18 gene expression in neutrophils. In bacterial infections, lipopolysaccharide from bacteria induces TREM-1 expression in neutrophils (Ramanathan et al., 2005). However, the specific ligand(s) for TREM-1 in *Leishmania* are unknown, thus warranting further investigations. Carneiro et al. found that high INFγ-producing individuals expressed genes associated with pathways related to TREM-1 signaling (Carneiro et al., 2016). In this case, the involvement of TREM-1 preferentially in high INFγ-producing individuals suggests that a stronger *Leishmania* response may be involved in tissue destruction and lesion development (Carneiro et al., 2016).

Neutrophil exposure to *L. infantum* reduced surface TREM-1 expression and increased sTREM-1 levels. Previous studies have reported that patients with sepsis had increased sTREM-1 and decreased membrane-bound TREM-1 expression on neutrophils compared to non-infectious systemic inflammatory response syndrome (Oku et al., 2013). Although we showed an increase in the TREM-1 gene expression, the elevated levels were not found on the neutrophil surface. This may be due to either TREM-1 surface shedding and/or alternative splicing (Baruah et al., 2015). In this study, we demonstrated increased release of sTREM-1 after *L. infantum* infection in a dose-response and time course effect. These results suggest that neutrophils are a major source of sTREM-1 in VL.

TREM-1 surface shedding may occur either by the action of neutrophil proteinases or proteases released by *Leishmania* (Mottram et al., 2004). Alternatively, it is possible that the presence of *Leishmania* induces the release of TREM-1 present in granules by alternative splicing (Baruah et al., 2015). One of the main pathways of recognition of *Leishmania* is the TLR2 pathway. Pathogen recognition by this pathway has been shown to induce expression of TREM-1 (Arts et al., 2013). Recently, Sacramento et al. (2017) showed that during *L. infantum* infection, TLR2 signaling has a direct effect on neutrophils, mediating their activation, NO and TNF production and *L. infantum* uptake. These results demonstrate that TLR2 signaling plays an important role in immune protection against *L. infantum* infection (Sacramento et al., 2017). Thus, reduction of TREM-1 on the surface of neutrophils may favor the establishment of the parasite, since signaling is important for the activation of microbicide mechanisms and induction of pro-inflammatory cytokines. Yizengaw et al. (2016) reported that neutrophils from patients with VL are highly activated. However, these patients also have an increased frequency of immature neutrophils that have impaired effector function, degranulation events, ROS production and phagocytic abilities (Yizengaw et al., 2016). These authors suggest that these dysfunctional neutrophils play a role in the systemic inflammatory response characteristic of VL patients and contribute to disease severity.

As sTREM-1 negatively regulate TREM-1 receptor signaling via ligand neutralization (Klesney-Tait et al., 2006), the increased sTREM-1 levels observed in severe VL, in which an exaggerated inflammatory response is observed, may be an attempt to restrain excessive inflammation in VL. This response could be largely beneficial to the patient. However, in the present study, high levels of sTREM-1 is associated with clinical parameters of disease severity. Additionally, our data show that leishmania is activating TREM-1 transcription and release of sTREM-1 from the neutrophils. Therefore, it is possible that the release of sTREM-1 induced by leishmania inhibits the inflammatory pathway and contributes to the disease by impairing TREM-1 receptor signaling.

## Author contributions

Conceived and designed the experiments: LB, VB, AJ, RA, and TdM. Follow-up of patients: AS and RA. Performed the experiments: LB, LM, MS-F, NP, CC, and TdM. Analyzed the data: LB, NP, CC, DT, ML, VB, AJ, RA, and TdM. Contributed reagents/materials/analysis tools: ML, VB, AJ, RA, and TdM. Wrote the manuscript: LB, NP, ML, AJ, and TdM.

### Conflict of interest statement

The authors declare that the research was conducted in the absence of any commercial or financial relationships that could be construed as a potential conflict of interest.
